# Associations of clinical obesity with arterial stiffness and cerebral small vessel disease: a population-based study

**DOI:** 10.3389/fendo.2026.1793231

**Published:** 2026-06-02

**Authors:** Mengmeng Bai, Ying Hui, Jingjie Liu, Xiaoshuai Li, Ling Yang, Chunyu Ruan, Wenfei Zhang, Shaohua Zhou, Siyu Jia, Jianmin Qiao, Han Lv, Shouling Wu

**Affiliations:** 1 Department of Graduate School, Hebei North University, Zhangjiakou, Hebei, China; 2 Department of Radiology, Kailuan General Hospital, Tangshan, Hebei, China; 3 Department of Radiology, Beijing Friendship Hospital, Capital Medical University, Beijing, China; 4 Department of Cardiology, Kailuan General Hospital, Tangshan, Hebei, China; 5 Department of Chengde Medical University, Chengde, Hebei, China

**Keywords:** arterial stiffness, cerebral small vessel disease, clinical obesity, diabetes mellitus, hypertension

## Abstract

**Objectives:**

Based on the comprehensive indicator of clinical obesity, this study investigates its association with arterial stiffness and cerebral small vessel disease (cSVD).

**Methods:**

This cross-sectional study analyzed 2,011 adults from the Multimodal Imaging Study based on the Kailuan Cohort. From 2021 to 2024, participants underwent Brain magnetic resonance imaging scans and obesity assessments. We used logistic regression models to evaluate associations between clinical obesity, arterial stiffness, and cSVD, adjusting for age, sex, smoking history, drinking history, physical activity, glucose-lowering medication, lipid-lowering medication, homocysteine, C-reactive protein, and low-density lipoprotein cholesterolMediation analysis tested whether arterial stiffness mediated the obesity–cSVD relationship.

**Results:**

A total of 2,011 participants (49.8% male, mean age 53.7 ± 12.2 years) were included. Clinical obesity was significantly associated with a higher total burden of cSVD, and the strength of its association with specific imaging markers decreased in the following order: perivascular spaces (PVS), lacunar infarcts, white matter hyperintensities (WMH), and cerebral microbleeds (CMB). Arterial stiffness significantly mediated the association between clinical obesity and cSVD.

**Conclusions:**

Clinical obesity was significantly associated with cSVD, with its association varying across specific imaging markers. Arterial stiffness mediates this relationship, suggesting that targeting obesity and arterial stiffness may help prevent subclinical cerebrovascular damage.

## 1 Introduction

Cerebral small vessel disease (cSVD) is an age-related condition characterized by a marked increase in prevalence with advancing age (1–3). Epidemiological studies indicate that its prevalence reaches approximately 5% in adults over 50 years old, approaching 100% in those aged 90 and above (4–6). cSVD is not only a common condition in the elderly but also a risk factor for stroke and cognitive decline (7–9). Meta-analyses indicate that individuals with cSVD have a 3·43-fold increased risk of stroke, along with 27% and 48% higher risks of cognitive decline and dementia, respectively, compared to those without the condition (10, 11). This imposes a substantial burden on society and healthcare systems. Furthermore, with the accelerating aging of the population, the burden attributable to cSVD is projected to continue to rise (5). The imaging markers of cSVD include white matter hyperintensities (WMH), lacunar infarcts, cerebral microbleeds (CMB), and perivascular spaces (PVS) (12). These markers have distinct pathological mechanisms. WMH is generally considered to result from demyelination due to chronic ischemia (12, 13). Lacunar infarcts primarily arise from ischemic necrosis of small perforating arteries (13). CMB may be caused by increased vascular wall fragility due to microvascular atherosclerosis (14). PVS enlargement may be related to impaired glymphatic function. Identifying modifiable risk factors for cSVD is important for early prevention (3, 15).

Currently recognized risk factors for cSVD include both traditional factors such as hypertension, arterial stiffness, OSA, smoking, and physical inactivity, as well as individual metabolic factors including obesity and general dysregulation (5, 16–18). Prior researches linking obesity to cSVD have largely utilized BMI to define overweight or obesity (19). Yet, BMI cannot distinguish fat from muscle or bone, offering an imprecise measure of adiposity (20–22). To address this, The Lancet Diabetes & Endocrinology Commission proposed a new definition of clinical obesity in January 2025, which moves beyond simple anthropometry to incorporate structural and functional alterations in tissues and organs (23). Although clinical obesity is theoretically expected to increase the risk of cSVD, its association with cSVD under the new diagnostic standard remains unexplored. Therefore, we leveraged data from the META-KLS subcohort of the Kailuan Study to examine the relationship between clinical obesity and cSVD and its imaging markers, and to investigate the potential mediating role of peripheral arterial stiffness.

## 2 Methods

### 2.1 Participants

**Figure 1:**
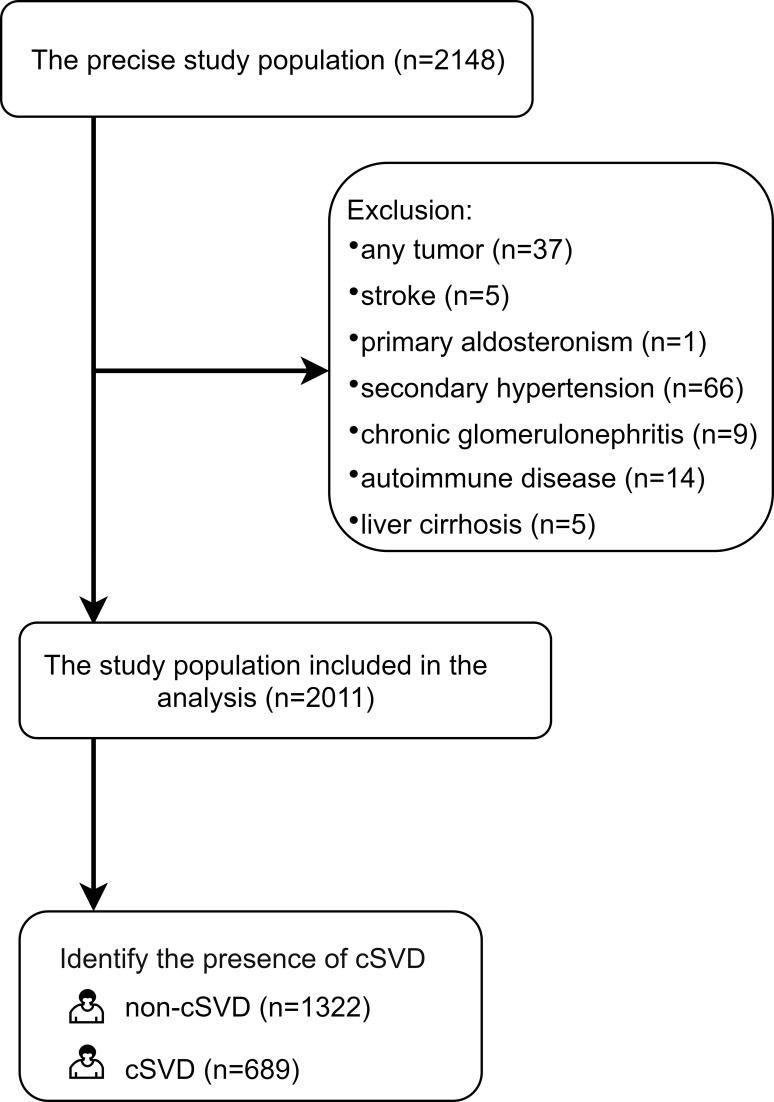
Flowchart of the study population.

META-KLS is an imaging subcohort embedded within the Kailuan Study—a prospective community-based cohort in Tangshan, China, initiated in 2006 with biennial follow-ups. To investigate the impact of multiple risk factors on brain health, META-KLS recruited participants from the parent cohort between December 2020 and December 2023. Enrolled participants underwent multimodal imaging (including brain MRI) and comprehensive clinical assessments within the ongoing follow-up framework. Participants were enrolled if they met all of the following criteria: (1) were existing members of the Kailuan Study cohort; (2) had anthropometric measurements (BMI, waist circumference, waist-to-hip ratio, waist-to-height ratio) sufficient for assessing clinical obesity; (3) had complete data required for clinical obesity diagnosis according to the 2025 Lancet Commission definition; and (4) provided informed consent for the META-KLS substudy and completed a brain MRI scan with interpretable data. Participants were excluded for any of the following: (1) history of brain tumor; (2) history of stroke; (3) diagnosis of primary aldosteronism (PA), secondary hypertension, chronic glomerulonephritis (CGN), autoimmune disease (AID), or liver cirrhosis. Participants with these conditions were excluded to avoid confounding effects from secondary blood pressure elevation or vascular inflammation. The prevalence of these conditions in the study population was low, and their exclusion does not affect sample representativeness. All participants provided written informed consent prior to enrollment **Figure 1**.

Ethical approval for both the Kailuan Study and the META-KLS subcohort was obtained from the Ethics Committee of Kailuan General Hospital (Approval Numbers: 2008 No. 1 and 2025013). All participants provided written informed consent upon enrollment.

### 2.2 Assessment of exposure variables

According to The Lancet Diabetes & Endocrinology Commission, clinical obesity is defined as a state of adiposity confirmed by anthropometric criteria and meeting at least one clinical criterion. These clinical criteria include evidence of organ or tissue dysfunction attributable to obesity, or significant age-adjusted limitations in daily activities that specifically reflect obesity’s impact on mobility and/or other essential activities of daily living. In contrast to clinical obesity, preclinical obesity is defined as a state of adiposity confirmed by anthropometric criteria but not meeting any of the clinical criteria. In this study, obesity status was determined through a comprehensive assessment of multidimensional data. The clinical criteria were adapted and prioritized according to the Kailuan Study data profile, as detailed in **Supplementary Table 1**.

#### 2.2.1 Assessment of obesity status

Anthropometric criteria were met if any of the following conditions were satisfied: (1) BMI ≥ 40 kg/m²; (2) BMI ≥ 28 kg/m² accompanied by an elevated value in any one of the following: waist circumference (>90 cm for men, >85 cm for women), waist-to-hip ratio (>0·90 for men, >0·85 for women), or waist-to-height ratio (>0·50 for both sexes); (3) Elevated values in any two of the following: waist circumference, waist-to-hip ratio, or waist-to-height ratio.

Trained staff collected anthropometric data (height, weight, waist circumference, and hip circumference) following standardized protocols. In the early phase, height was measured using a stadiometer, waist and hip circumferences with a tape measure, and weight with a floor scale. In later phases, an integrated stadiometer and scale instrument was adopted for uniform height and weight assessment. Body mass index (BMI) was calculated from height and weight, the waist-to-hip ratio from waist and hip circumferences, and the waist-to-height ratio from waist circumference and height.

#### 2.2.2 Assessment of variables related to clinical obesity diagnosis

Trained healthcare staff conducted face-to-face interviews with participants using a standardized questionnaire. Information collected included sex, history of snoring, limitations in daily activities, medical history (hypertension, dyslipidemia, diabetes, and headaches), and medication history (antihypertensive drugs). Following a standardized protocol, participants rested seated for 5 minutes after abstaining from smoking, tea, or coffee for ≥30 minutes. Right brachial blood pressure was then measured three times at 1–2 minutes intervals using an Omron HBP-1100U electronic monitor, with the average of the three readings calculated for analysis. Fasting blood samples were collected from the antecubital vein in the morning. Fasting blood glucose (FBG), triglycerides (TG), high-density lipoprotein cholesterol (HDL-C), and creatinine (CREA) were measured using an automatic analyzer (Hitachi 008AS) at the central laboratory of Kailuan General Hospital. Urine protein content was determined using urinalysis test strips (H12-MA). Relevant medical histories were collected from case documentation, encompassing COPD, obstructive sleep apnea (OSA), atrial fibrillation, heart failure, deep vein thrombosis, pulmonary embolism, pulmonary hypertension, non-alcoholic fatty liver disease with liver fibrosis, intracranial hypertension, chronic or severe knee or hip pain, and urinary incontinence.

#### 2.2.3 Assessment of potential covariates

Prior evidence suggests that certain confounding factors may influence the obesity-brain health relationship. Prior evidence suggests that certain confounding factors may influence the obesity-brain health relationship. These covariates, partially overlapping with the clinical obesity diagnostic variables mentioned above, were obtained via questionnaire and included age, drinking history, smoking history, physical activity, and medication history (glucose-lowering and lipid-lowering drugs). Laboratory measures such as homocysteine (HCY), C-reactive protein (CRP), and low-density lipoprotein cholesterol (LDL-C) were analyzed using automated instruments at the central laboratory of Kailuan General Hospital.

### 2.3 Assessment of outcome variables

#### 2.3.1 Image acquisition

All MRI scans were acquired using a 3·0T scanner (General Electric 750 W, Milwaukee, WI, USA). The imaging protocol comprised the following sequences: 3D T1-weighted BRAVO, 3D T2-weighted imaging, sagittal 3D T2-FLAIR, diffusion-weighted imaging (DWI), and susceptibility-weighted angiography (SWAN). The detailed acquisition parameters for all structural MRI sequences are summarized in supplementary **Supplementary Table 2**.

#### 2.3.2 Assessment of cSVD

Imaging markers of cSVD included lacunar infarcts, WMH, CMBs, and PVS. The total cSVD burden was assessed using the MRI-based global burden score proposed by Staals et al. This composite score integrates the four individual imaging markers into a single ordinal scale ranging from 0 to 4 (24, 25). According to this criterion, one point was added to the total score for each of the following criteria: (1) presence of any lacunar infarct; (2) presence of any CMB; (3) moderate-to-severe (>10) visible PVS in the basal ganglia; and (4) a Fazekas score of 3 for periventricular WMH and/or a Fazekas score ≥2 for deep WMH. Consequently, the total cSVD burden score ranges from 0 to 4, with each marker contributing a binary score of 0 or 1. In this study, a total cSVD burden score of ≥1 served as the cutoff to define the presence of cSVD and was used as the primary endpoint. Inter-rater reliability for cSVD total burden and individual marker scores was assessed using Cohen’s kappa (κ). The κ coefficients were as follows: 0·784 for lacunar infarcts, 0·869 for WMH, 0·878 for CMB, 0·715 for PVS, and 0·703 for the total cSVD burden, indicating good to excellent agreement.

### 2.4 Assessment of mediating variables

#### 2.4.1 Assessment of Brachial-Ankle Pulse Wave Velocity

baPWV was assessed with the BP-203 RPE III arteriosclerosis detector. All measurements were conducted by trained personnel before MRI under a standardized protocol. Participants were required to avoid smoking and to rest supine for at least 5 minutes in a room maintained at 22–25 °C. Cuff placement was standardized (arm: 2–3 cm above cubital fossa; ankle: 1–2 cm above medial malleolus), and a heart sound detector was positioned at the left sternal border. Two consecutive measurements were obtained for each participant, with the second reading used for analysis.

#### 2.4.2 Definition of arterial stiffness

Arterial stiffness was categorized based on the higher (left or right) baPWV value for analysis. It was defined as follows: normal range, baPWV < 1400 cm/s; mild arterial stiffness, 1400 ≤ baPWV < 1800 cm/s; and moderate-to-severe arterial stiffness, baPWV ≥ 1800 cm/s (26, 27).

### 2.5 Statistical analysis

This study followed the STROBE guidelines. All analyses were performed using SAS 9·4, with a two-sided P < 0·05 considered significant. The sample size calculation was based on a conservative estimate using the cSVD prevalence in the normal group and the clinical obesity group (α=0.05, two-sided, power=0.80). Given the small sample size of the pre-clinical obesity group (n=317), the calculation was performed only for the normal and clinical obesity groups to ensure adequate statistical power for the primary comparison.

Categorical variables are expressed as counts (percentages). Continuous variables are expressed as mean ± SD if normally distributed, or median (IQR) otherwise. Group comparisons used the χ² test, Wilcoxon rank-sum test, or Student’s t-test, as appropriate. To assess the strength of associations, we employed the following models: ordinal logistic regression was used to analyze the associations of clinical obesity with total cSVD burden and with the degree of arterial stiffness, while binary logistic regression was used to analyze its associations with individual cSVD imaging markers. All associations are reported as odds ratios (ORs) with 95% confidence intervals (CIs). To account for potential confounding, we constructed three sequential models. Model 1 was adjusted for age and sex. Model 2 additionally included drinking history, smoking history, and physical activity. Model 3 further incorporated LDL-C, CRP, homocysteine HCY, and use of lipid-lowering medications. Missing covariate data were handled using multiple imputation by chained equations. The imputed variables included smoking history, drinking history, physical activity, LDL-C, HCY and CRP. Five imputed datasets were generated using the fully conditional specification method, with the maximum number of iterations set to 200 to ensure that all parameter estimates reached standard convergence levels.

A mediation analysis was conducted to test whether arterial stiffness mediates the link between clinical obesity and cSVD. We utilized the SAS CAUSALMED procedure, which relies on asymptotic maximum likelihood theory and a bootstrap method (1,000 resamples) to estimate the direct (unmediated), indirect (mediated), and total effects, adjusting for the full set of covariates from Model 3: age, sex, drinking history, smoking history, physical activity, LDL-C, CRP, HCY, and lipid-lowering medication use.

To assess the robustness of our findings, we conducted a series of sensitivity analyses. We performed two sets of stratified analyses to assess the association between clinical obesity and cSVD (total score ≥1) using binary logistic regression (OR, 95% CI). One set stratified participants by the number of clinical criteria (1 vs. ≥2). The other set consisted of subgroup analyses by age (<55 vs. ≥55 years) and by sex.

In a further analysis, we modified the dichotomization of the total cSVD burden score by defining moderate-to-severe cSVD as a score of ≥2. Using this definition, we employed binary logistic regression to calculate the odds ratio (OR) and 95% confidence interval (CI) for the association between clinical obesity and moderate-to-severe cSVD.

Effect estimates are presented with 95% confidence intervals. Odds ratios are reported to two decimal places. Given the scale of mediation effect estimates, they are presented to three decimal places.

## 3 Results

### 3.1 Baseline characteristics

Of the 2,141 participants initially enrolled, those meeting any of the following criteria were excluded: tumors (n=37, 1.73%), stroke (n=5, 0.23%), PA (n=1, 0.05%), secondary hypertension (n=66, 3.08%), CGN (n=9, 0.42%), AID (n=14, 0.65%), and liver cirrhosis (n=5, 0.23%). Because some participants met multiple exclusion criteria, 130 participants were excluded, leaving 2,011 participants in the final analysis.

**Table 1** summarizes the baseline characteristics of the study population according to the presence or absence of cSVD. Among the 2,011 participants, 1,002 (49·83%) were male, with a mean age of 53·66 ± 12·22 years. A total of 689 (34·26%) were classified as non-cSVD, and 1,322 (65·74%) were in the cSVD group. Participants with cSVD were older and had higher SBP, FBG, LDL-C, CRP, and HCY levels, but lower HDL-C levels, compared to those without cSVD. They also had a higher prevalence of hypertension, diabetes, dyslipidemia, smoking history, drinking history, and physical activity (Baseline characteristics stratified by clinical obesity status are presented in **Supplementary Table 3**).

**Table 1:** Baseline characteristics of participants.

Characteristics	Total (n=2011)	Non-cSVD (n=689)	cSVD (n=1322)	*P* value
Age, years	53.66 ± 12.22	43.37 ± 9.34	59.03 ± 9.89	<0.01 ^a^
Male, n%	1002 (49.83)	248 (35.99)	754 (57.03)	<0.01 ^c^
BMI, kg/m²	25.11 ± 3.54	24.79 ± 3.81	25.27 ± 3.37	<0.01 ^a^
SBP, mmHg	132.06 ± 19.12	123.46 ± 16.51	136.54 ± 18.86	<0.01 ^a^
DBP, mmHg	78.92 ± 12.23	76.55 ± 12.10	80.16 ± 12.12	<0.01 ^a^
FBG, mmol/L	5.58 ± 1.31	5.26 ± 0.87	5.74 ± 1.46	<0.01 ^a^
LDL-C, mmol/L	3.13 ± 0.79	3.02 ± 0.75	3.18 ± 0.80	<0.01 ^a^
HDL-C, mmol/L	1.47 ± 0.35	1.50 ± 0.37	1.46 ± 0.34	0.02 ^a^
TG, mmol/L	1.68 ± 1.34	1.58 ± 1.33	1.74 ± 1.34	<0.01 ^a^
Hypertension, n%	1042 (51.82)	204 (29.61)	838 (63.39)	<0.01 ^c^
Diabetes mellitus, n%	240 (11.93)	35 (5.08)	205 (15.51)	<0.01 ^c^
Hyperlipemia, n%	896 (44.55)	239 (34.69)	657 (49.70)	<0.01 ^c^
Antihypertensive treatment, n%	652 (32.42)	95 (13.79)	557 (42.13)	<0.01 ^c^
Hypoglycemic treatment, n%	146 (7.26)	15 (2.18)	131 (9.91)	<0.01 ^c^
Lipid-lowering treatment, n%	246 (12.23)	85 (9.74)	18 (5.68)	<0.01 ^c^
Smoking history, n%	587 (29.19)	136 (19.74)	451 (34.11)	<0.01 ^c^
Drinking history, n%	728 (36.20)	187 (27.14)	541 (40.92)	<0.01 ^c^
Physical exercise, n%	1154 (57.38)	308 (44.70)	846 (63.99)	<0.01 ^c^
HCY, μmol/L	12.49 (10.22-15.86)	11.04 (9.23-13.84)	13.18 (10.90-16.81)	<0.01 ^b^
CRP, mg/L	1.20 (0.60-2.30)	1.10 (0.50-2.20)	1.30 (0.70-2.30)	<0.01 ^b^
Obesity status				<0.01 ^c^
Non-Clinical obesity	873 (43.41)	367 (53.27)	506 (38.28)	
Preclinical obesity, n%	317 (15.76)	154 (22.35)	163 (12.33)	
Clinical obesity, n%	821 (40.83)	168 (24.38)	653 (49.39)	
WMH, n%	439 (21.83)	0	439 (33.21)	<0.01 ^c^
PVS, n%	1183 (53.83)	0	1183 (89.49)	<0.01 ^c^
LA, n%	276 (13.72)	0	276 (20.88)	<0.01 ^c^
CMB, n%	506 (25.16)	0	506 (38.28)	<0.01 ^c^

Data are presented as the mean ± standard deviation, median (interquartile range), or number (percentage). BMI, body mass index; SBP, systolic blood pressure; DBP, diastolic blood pressure; FBG, fasting blood glucose; LDL-C, low density lipoprotein cholesterol; HDL-C, high density lipoprotein cholesterol; TG, triglyceride; HCY, homocysteine; CRP, C-Reactive Protein; PVS, perivascular spaces; CMB, cerebral microbleeds; LA: lacunar infarcts; WMH, white matter hyperintensities. ^a^One-way analysis of variance. ^b^Wilcoxon rank-sum test. ^c^Chi-squared test.

### 3.2 Association between clinical obesity and cSVD

As shown in **Table 2**, ordinal logistic regression analysis revealed that, with reference to the normal group and after sequential adjustment for confounders, individuals with clinical obesity had a significantly higher risk of cSVD, with an odds ratio (OR) of 1·48 (95% CI: 1·22–1·80).

**Table 2:** Association of obesity status and cerebral small vessel disease burden.

Group	No. of cases/Total	Model 1		Model 2		Model 3	
		OR (95%CI)	*P*	OR (95%CI)	*P*	OR (95%CI)	*P*
Non-obesity	506/873	1		1		1	
Preclinical obesity	163/317	0.67 (0.51-0.87)	<0.01	0.66 (0.51-0.86)	<0.01	0.67 (0.52-0.88)	<0.01
Clinical obesity	652/821	1.60 (1.32-1.93)	<0.01	1.59 (1.31-1.92)	<0.01	1.48 (1.22-1.80)	<0.01

Ordinal Logistic Regression Analysis is used to investigate the association of Obesity Status and Cerebral Small Vessel Disease Burden. OR, Odds Ratio; CI, confidence interval.Model 1: adjusted for age and gender;Model 2: adjusted for variables in Model 1 plus smoking (yes or no), drinking (yes or no), Physical exercise (yes or no);Model 3: adjusted for variables in Model 2 plus LDL, (mmol/L), HCY (umol/L), CRP (mg/L), Hypoglycemic treatment (yes or no), Lipid-lowering treatment (yes or no).

**Figure 2:**
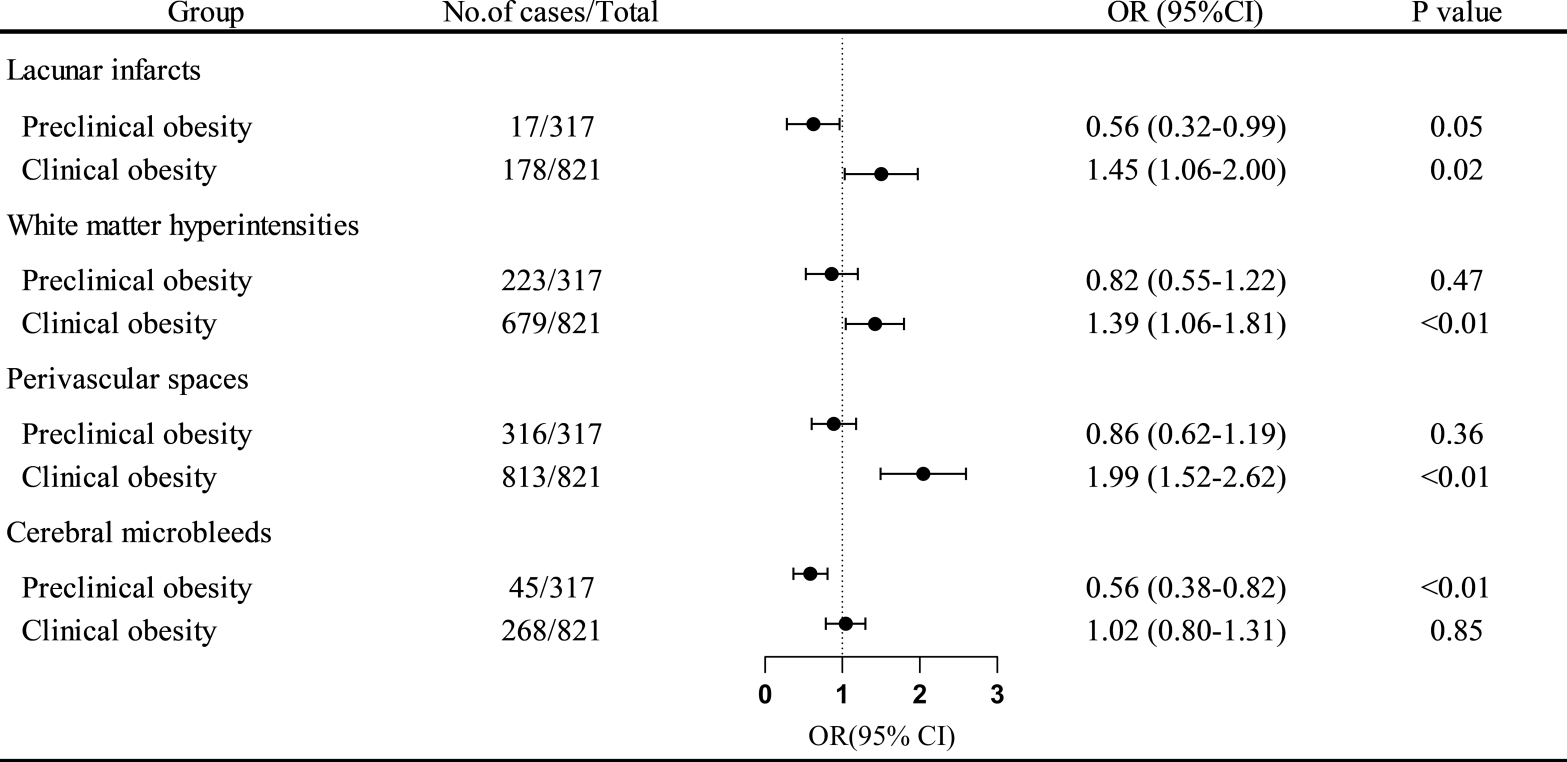
Association between clinical obesity and cerebral small vessel disease markers: A forest plot analysis using binary logistic regression. OR, odds ratio; CI, confidence interval.

As shown in **Figure 2**, after adjustment for multiple confounders, binary logistic regression analyses indicated that the clinical obesity group had significantly higher risks of lacunar infarcts (OR 1·45, 95% CI 1·06–2·00), WMH (OR 1·39, 95% CI 1·06–1·81), and enlarged PVS (OR 1·99, 95% CI 1·52–2·62) compared to the normal group. No significant association was observed for CMBs. Furthermore, the preclinical obesity group exhibited no significantly elevated risk for any cSVD imaging marker.

### 3.3 Association between clinical obesity and arterial stiffness

Compared to the normal group, participants with clinical obesity had a significantly higher risk of arterial stiffness (OR 1·93, 95% CI 1·54–2·43), as indicated by ordinal logistic regression analysis after adjustment for multiple confounders, which established clinical obesity as an independent risk factor (**Supplementary Table 4**).

### 3.4 Association between arterial stiffness and cSVD

Adjusted ordinal logistic regression analysis revealed a significantly elevated risk of cSVD in the moderate-to-severe arterial stiffness group compared to the normal group (OR 2·03, 95% CI 1·04–4·17). In contrast, no significant association was observed for the mild stiffness group (**Supplementary Table 5**).

**Figure 3:**
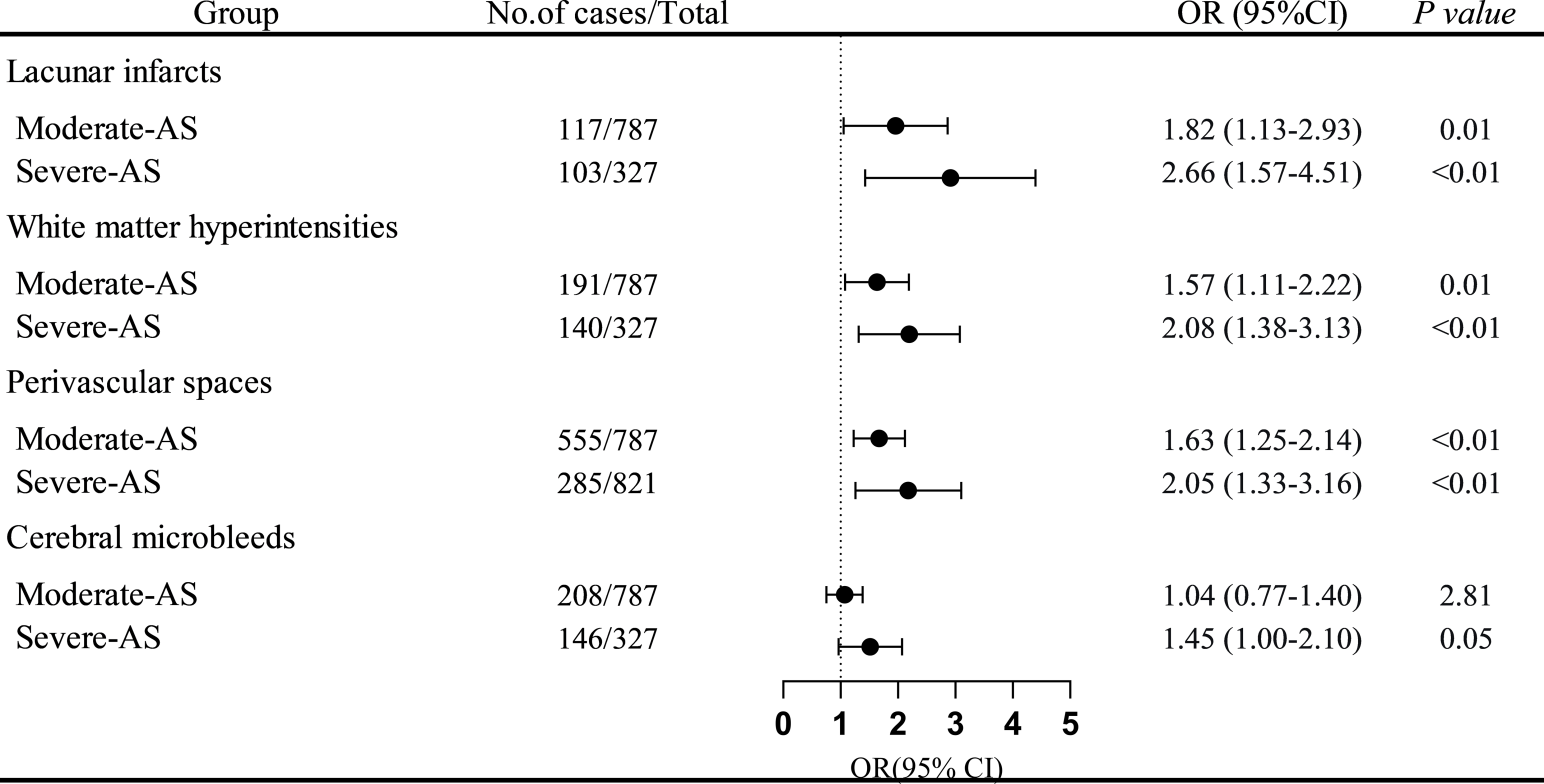
Association between arterial stiffness and cerebral small vessel disease markers: A forest plot analysis using binary logistic regression. OR, odds ratio; CI, confidence interval; AS, arterial stiffness.

Binary logistic regression analysis, after adjusting for multiple confounders, demonstrated that compared to the normal group, both arterial stiffness groups had significantly increased risks for specific cSVD markers. The mild arterial stiffness group showed higher risks for lacunar infarcts (OR 1·82, 95% CI 1·13–2·93), WMH (OR 1·57, 95% CI 1·11–2·22), and enlarged PVS (OR 1·63, 95% CI 1·25–2·14). Similarly, the moderate-to-severe arterial stiffness group exhibited elevated risks for lacunar infarcts (OR 2·66, 95% CI 1·57–4·51), WMH (OR 2·08, 95% CI 1·38–3·13), and enlarged PVS (OR 2·05, 95% CI 1·33–3·16). In contrast, neither stiffness group showed a statistically significant difference in CMB burden compared to the normal group (**Figure 3**).

### 3.5 Analysis of mediation

**Figure 4:**
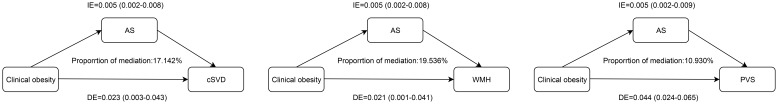
Mediation effect by arterial stiffness in the association between Clinical obesity and cSVD burden and its imaging markers. DE, direct effect; IE, indirect effect; AS, arterial stiffness; cSVD, cerebral small vessel disease; WMH, white matter hyperintensities; PVS, enlarged perivascular spaces.

The mediation analysis based on the fully adjusted Model 3 (**Figure 4**) demonstrated that arterial stiffness significantly mediated the associations of clinical obesity with total cSVD burden (direct effect: 0·023 [95% CI 0·003–0·043]; indirect effect: 0·005 [95% CI 0·002–0·008]; proportion mediated: 17·14%), with WMH (direct effect: 0·021 [95% CI 0·001–0·041]; indirect effect: 0·005 [95% CI 0·002–0·008]; proportion mediated: 19·54%), and with enlarged PVS (direct effect: 0·044 [95% CI 0·024–0·065]; indirect effect: 0·005 [95% CI 0·002–0·009]; proportion mediated: 10·93%).

### 3.6 Sensitivity Analyses

In sensitivity analyses, the strength of the association between clinical obesity and cSVD varied by the number of concomitant clinical criteria. A significant association was present for individuals with one criterion (OR 1·44, 95% CI 1·03–2·01), with a stronger association observed for those with two or more criteria (OR 1·80, 95% CI 1·13–2·85) (**Supplementary Table 6**).

**Figure 5:**
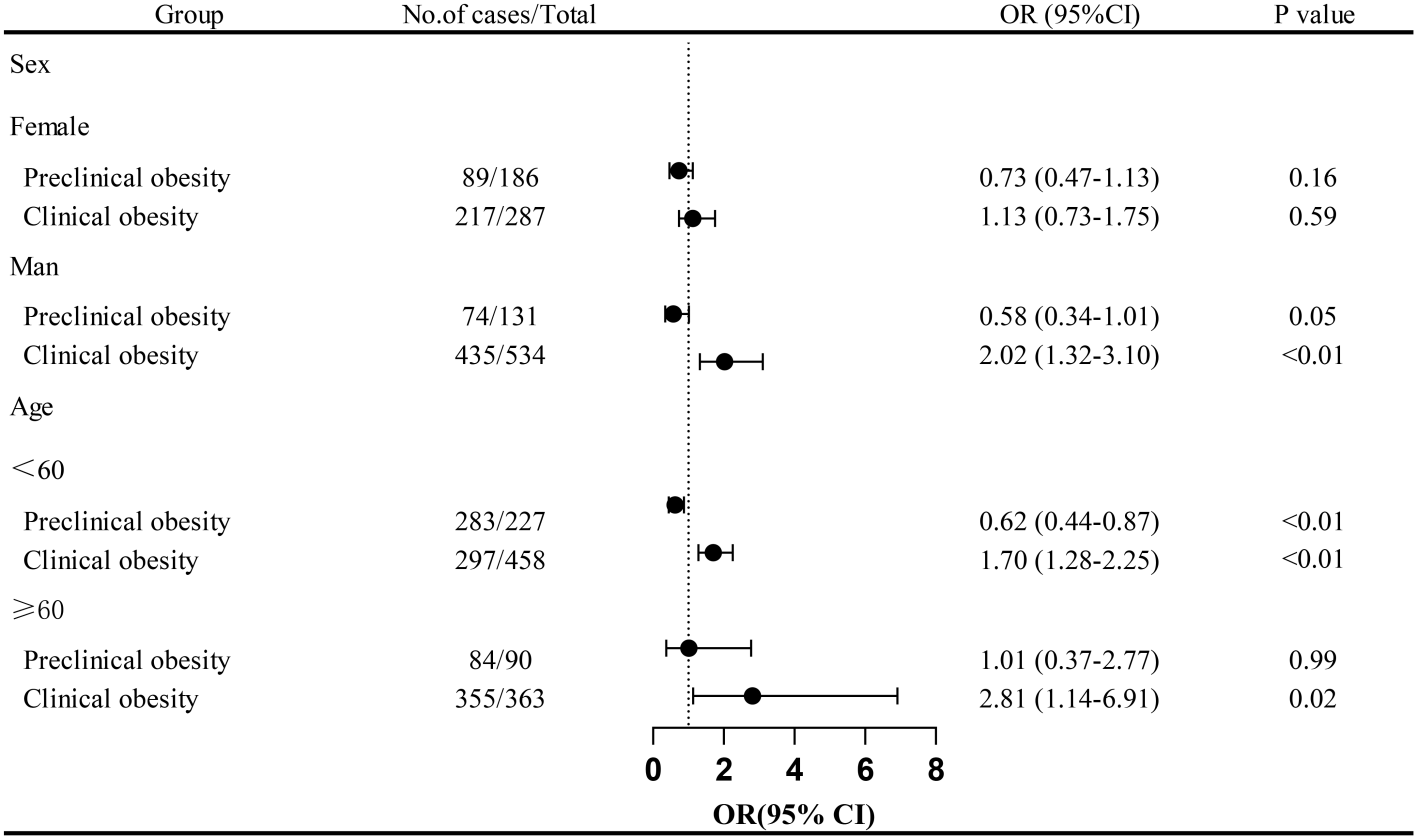
Association between clinical obesity and cerebral small vessel disease: A forest plot of binary logistic regression analyses stratified by sex and age. OR, odds ratio; CI, confidence interval.

Further sensitivity analyses stratified by sex and age revealed notable effect modifications. Sex-stratified analysis indicated a significant gender difference: the association between clinical obesity and cSVD was significant in males (OR 2·02, 95% CI 1·32–3·10) but not in females, relative to their respective normal groups. Age-stratified analysis showed that the association was significant in both age groups, with a stronger effect observed in participants aged ≥60 years (OR 2·81, 95% CI 1·14–6·91) than in those <60 years (OR 1·70, 95% CI 1·28–2·25) (**Figure 5**).

Finally, clinical obesity was significantly associated with moderate-to-severe cSVD (OR 1·64, 95% CI 1·26–2·13) (**Supplementary Table 7**).

## 4 Discussion

This study found that clinical obesity is significantly associated with cSVD. Furthermore, the strength of this association depends on the specific underlying pathology, with the strength of association descending in the order of PVS, lacunar infarcts, WMH, and CMB. Arterial stiffness mediated the associations between clinical obesity and cSVD, PVS, and WMH. Furthermore, the association between clinical obesity and cSVD was modified by sex and age.

Previous studies on obesity defined by BMI and cSVD have not reached consistent conclusions. Longitudinal data from the UK Biobank provide genetic evidence that predisposition to elevated BMI is linked to higher cSVD burden (28). These data further show that phenotypically higher BMI is correlated with increased WMH load and volume (21, 29). In an analysis of the Japanese Brain Dock cohort, Ishida et al. observed no significant link of total cSVD burden with either isolated abdominal obesity or general obesity (30). This study found that clinical obesity was significantly associated with cSVD, conferring a 48% higher risk compared to the normal group. Compared to previous studies, our findings are more reliable for two reasons. First, we applied a more precise method to define clinical obesity, which excluded individuals with elevated BMI due to high muscle or bone mass rather than adiposity. Second, our criteria incorporated obesity-related alterations in tissue and organ structure and function.

This study establishes a significant positive association between clinical obesity and cSVD, and further reveals—as a novel finding—that the strength of this association is pathology-specific. The risk hierarchy is defined by the following odds ratios: highest for PVS (OR 1·99), intermediate for lacunar infarcts (OR 1·45) and WMH (OR 1·39), and absent for CMB. Despite searching major databases, we found no studies that systematically compared how obesity associates differently with individual cSVD imaging components. In a cross-sectional analysis of the Swedish Kungsholmen Aging and Care Study, Laveskog et al. reported no significant association between obesity and global or regional PVS (31). Drawing on the same health examination database at Seoul National University Hospital, Nam et al. reported that a higher A Body Shape Index (ABSI) was associated with increased WMH volume and a 41% greater risk of lacunar infarcts; separately (32), Kim et al. found that BMI-defined overweight was associated with a 117% elevated risk of deep or infratentorial microbleeds (33). These findings suggest that the strength of the association between obesity and individual cSVD components exhibits component-specific variation.

On one hand, the heterogeneity in these associations likely reflects distinct underlying mechanisms. We hypothesize that the association between clinical obesity and PVS is primarily driven by three core factors: hypertension, metabolic dysregulation, and OSA (5, 9, 34). These conditions collectively contribute to blood-brain barrier dysfunction and impaired glymphatic drainage, thereby promoting PVS formation (35–37). In our study population, all three comorbidities were notably more prevalent in the clinical obesity group. Hypertension and atrial fibrillation are likely risk factors for lacunar infarcts. The former may lead to vessel occlusion via penetrating artery disease, whereas the latter can cause lacunar infarcts through microemboli blocking the same penetrating arteries (38–40). In contrast, WMH, which represent diffuse white matter injury, are more closely linked to conditions that cause systemic hypoperfusion—such as heart failure and atrial fibrillation—and to those that induce chronic hypoxia, including chronic obstructive pulmonary disease and OSA (41–43). Hypertension may impair cerebral autoregulation, indirectly leading to a state of cerebral hypoperfusion (9, 44), which could explain why the strength of its association with WMH is second only to that with lacunar infarcts. The association between obesity and CMB is not uniformly distributed throughout the brain. Lobar CMBs are more closely linked to cerebral amyloid angiopathy (CAA), suggesting that obesity-related microbleeds may predominantly occur in deep or infratentorial regions (45). Furthermore, in our study population, anticoagulant use—a key intervention for atrial fibrillation, a major risk factor for CMBs—was relatively low.

On the other hand, from the perspective of the dynamic progression of cSVD, the pathological stage at which different imaging markers manifest influences the strength of their association with clinical obesity. As a key conduit for fluid and waste clearance in the brain, the PVS may be the earliest and most persistently affected structure by clinical obesity (35). This supports the view that PVS enlargement serves as an early and sensitive biomarker of cSVD (46). WMH and lacunar infarcts represent intermediate-to-advanced, irreversible structural damage. WMH results from the cumulative effects of chronic hypoperfusion and hypoxia, while lacunar infarcts arise from the occlusion of penetrating arteries (47). The difference in the strength of their associations suggests that the appearance of lacunar infarcts may indicate a transition into a more severe phase of obesity-related cerebrovascular pathology. Although both lacunar infarcts and CMB represent terminal outcomes following acute events, our results indicate that the pathological process driven by clinical obesity in this cohort is more likely to culminate in the ischemic lesion—lacunar infarcts—rather than the hemorrhagic lesion—CMB.

Previous studies have demonstrated that the major components of clinical obesity are all risk factors for arterial stiffness (23), which is known to cause the most pronounced damage to low-resistance, high-flow organs and to be a significant contributor to cSVD (48, 49). This study confirms prior findings, but differs from previous research in that we further demonstrate that arterial stiffness acts as a mediator in the associations between clinical obesity and both cSVD (mediation proportion: 17·14%), PVS (10·93%), and WMH (19·53%). These findings suggest that assessing arterial stiffness in the general population or in individuals with clinical obesity may help identify those at higher risk for cSVD. The potential value of clinical obesity and arterial stiffness for cSVD risk stratification warrants further investigation in longitudinal studies.

Sensitivity analyses further validated the robustness of the main findings. Clinical obesity accompanied by two or more clinical criteria was associated with a higher cSVD risk compared to obesity with only one criterion, indicating that the accumulation of clinical symptoms exacerbates brain health risk. This underscores the importance of strictly controlling the number of concomitant clinical symptoms while managing the obese state itself. Stratified analyses revealed a significant sex-specific difference(interaction *P* = 0.04): the association between clinical obesity and cSVD was significant in males but not in females. This discrepancy may be attributed to the relatively lower proportion of clinically obese women in our cohort and/or to the potential protective effects of estrogen on vasculature, which could delay the progression of cSVD pathology. The obesity-associated cSVD risk was nearly two-fold higher among individuals aged ≥60 years (OR 2·81) versus those <60 years (OR 1·70). However, the test for interaction was not statistically significant(interaction *P* = 0.75), indicating insufficient evidence to conclude that age is a significant effect modifier in this cohort. Larger sample sizes are needed to validate the potential synergistic role of age.

This study has several strengths. First, brain imaging was evaluated by radiologists, and clinical obesity was used as a more precise variable than traditional anthropometric measures. Second, we systematically examined the associations of clinical obesity with cSVD and its individual markers, and further analyzed the relationships among clinical obesity, arterial stiffness, and cSVD. These findings provide more comprehensive evidence for understanding the pathological process of cSVD. This study also has several limitations. The pre-clinical obesity group had a small sample size and comprised mostly young and middle-aged adults, and the corresponding estimates may be unstable. Second, the cross-sectional design precludes causal inference. The relationships among clinical obesity, arterial stiffness, and cSVD await validation in longitudinal studies.

In summary, our study leverages the detailed brain MRI phenotyping of the META-KLS cohort and a refined definition of clinical obesity to demonstrate its adverse impact on cSVD in Chinese adults. We show that clinical obesity is independently linked to higher risks of PVS, lacunar infarcts, and WMH, and additionally identify arterial stiffness as a mediator in the pathways linking obesity to cSVD, WMH, and PVS. These findings indicate that evaluating clinical obesity and its metabolic components, together with assessing arterial stiffness, may help identify individuals at high risk for subclinical brain structural injury.

## Data Availability

The original contributions presented in the study are included in the article/**Supplementary Material**. Further inquiries can be directed to the corresponding author.
