# Supplementary material for: Associations of clinical obesity with arterial stiffness and cerebral small vessel disease: a population-based study
Source: Front Endocrinol (Lausanne). 2026 Jun 2;17:1793231. doi: 10.3389/fendo.2026.1793231 (PMC13268931; doi:10.3389/fendo.2026.1793231)
Supplement: Supplementary file 1 [file Table1.docx]

Supplementary Material

# Supplementary Tables

| Cardiovascular System (Arterial) | SBP ≥ 140 mmHg or DBP ≥ 90 mmHg, use of any antihypertensive medication, or self-reported history of hypertension. |
| --- | --- |
| Metabolism | Hyperglycemia: FBG ≥ 6.1 mmol/L (≥ 110 mg/dL) or a confirmed diagnosis of type 2 diabetes.  Hypertriglyceridemia: TG ≥ 1.7 mmol/L (≥ 150 mg/dL).  Low HDL cholesterol: HDL-C < 1.0 mmol/L (< 40 mg/dL). |
| Upper Respiratory Tract | Diagnosis of OSA or report of habitual snoring |
| Respiratory System | Diagnosis of COPD |
| Cardiovascular System (Atrial) | Diagnosis of atrial fibrillation |
| Cardiovascular System (Ventricular) | Diagnosis of heart failure |
| Cardiovascular (Thrombotic) | Diagnosis of DVT and/or pulmonary embolism |
| Renal | eGFR was calculated using the CKD-EPI equation. It was defined as eGFR < 60 mL/min/1.73m² combined with a urine dipstick result of ≥1+ for proteinuria. |
| Cardiovascular System (Pulmonary) | The diagnosis of pulmonary hypertension was obtained from the echocardiography database. PASP was estimated by measuring tricuspid regurgitation velocity on echocardiography, with PASP ≥ 40 mmHg suggesting possible pulmonary hypertension. |
| Musculoskeletal System | Chronic, severe pain of the knee or hip, accompanied by joint stiffness and reduced range of motion, excluding cases attributed to rheumatoid arthritis, osteoarthritis, or prior hip arthroplasty due to trauma. |
| Hepatic | NAFLD with hepatic fibrosis: NAFLD was diagnosed based on hepatic ultrasonography and assessment of alcohol consumption. Hepatic fibrosis was evaluated using the FIB-4 score, with a threshold of ≥3.25 indicating significant hepatic fibrosis or cirrhosis. |
| Central Nervous System | Diagnosis of increased intracranial pressure or recurrent headache symptoms, excluding concurrent acute cerebral infarction, intracerebral hemorrhage, traumatic brain injury, or encephalitis. |
| Urinary System | Diagnosis of urinary incontinence. |
| Limitations in Daily Activities | Functional limitations were assessed using the ADL questionnaire. |

**Supplementary table S1.** Clinical criteria (re-sorted based on the characteristics of the Kailuan Study data). Abbreviations: Sbp, systolic blood pressure; DBP, diastolic blood pressure; FBG, fasting plasma glucose; TG, Triglyceride; HDL-C, HDL cholesterol; OSA, obstructive sleep apnea; COPD, chronic obstructive pulmonary disease; DVT, deep vein thrombosis; PE, pulmonary embolism; eGFR,estimated glomerular filtration rate; CKD-EPI, Chronic Kidney Disease Epidemiology Collaboration; PASP, pulmonary artery systolic pressure; NAFLD, Non-alcoholic fatty liver disease; ADL, activities of daily living.

| Sequence | TR (ms) | TE (ms) | Flip Angle (degree) | Slice Thickness (mm) | Gap (mm) | FOV (mm) | Matrix | Number of Slices |
| --- | --- | --- | --- | --- | --- | --- | --- | --- |
| 3D T1 BRAVO | 6.7 | 2.6 | 15 | 1.0 | 1.0 | 256 × 256 | 256 × 256 | 170 |
| 3D T2WI | 2500 | 84.9 | 90 | 1.0 | 1.0 | 256 × 256 | 256 × 256 | 166 |
| Sagittal 3D T2 FLAIR | 5000 | 1147 | 90 | 1.0 | 1.0 | 256 × 256 | 256 × 256 | 170 |
| DWI | 5110 | 77.2 | 90 | 5.0 | 6.0 | 240 × 240 | NA | 25 |
| SWAN | 37.9 | 23.9 | 15 | 2.0 | 1.0 | 240 × 240 | NA | 136 |

**Supplementary table S2.** Magnetic Resonance Imaging Acquisition Parameters. Abbreviations: DWI, diffusion-weighted imaging; FLAIR, fluid-attenuated inversion recovery; FOV, field of view; SWAN, susceptibility weighted angiography; TE, echo time; TR, repetition time; (not applicable in these sequences, marked as “NA”).

| **Table S3 Baseline characteristics of participants** | | | | | |
| --- | --- | --- | --- | --- | --- |
| Characteristics | Total (n=2011) | Non-obesity (n=873) | Preclinical obesity (n=317) | Clinical obesity (n=821) | P value |
| Hypertension, n% | 1042 (51.82) | 312 (35.74) | 0 | 730 (88.92) | <0.01 |
| Metabolic Disorders, n% | 21 (1.04) | 1 (0.11) | 0 | 20 (2.44) | <0.01 |
| Chronic Obstructive Pulmonary Disease, n% | 2 (0.10) | 2 (0.23) | 0 | 0 | <0.01 |
| Apnoeas/hypopnoeas during sleep, n% | 281 (13.97) | 74 (8.48) | 0 | 207 (25.21) | <0.01 |
| Recurrent headache, n% | 259 (12.88) | 68 (7.79) | 0 | 191 (23.26) | <0.01 |
| Atrial fibrillation, n% | 68 (3.38) | 19 (2.18) | 0 | 49 (5.97) | <0.01 |
| Heart Failure, n% | 10 (0.50) | 4 (0.46) | 0 | 6 (0.73) | 0.28 |
| Pulmonary artery hypertension, n% | 53 (2.64) | 28 (3.21) | 0 | 25 (3.05) | <0.01 |
| Recurrent DVT and/or pulmonary thromboembolic disease, n% | 2 (0.10) | 0 | 0 | 2 (0.24) | 0.23 |
| NAFLD with hepatic fibrosis, n% | 55 (2.73) | 8 (0.92) | 0 | 47 (5.72) | <0.01 |
| Microalbuminuria with reduced eGFR, n% | 3 (0.15) | 1 (0.11) | 0 | 2 (0.24) | 0.60 |
| Recurrent/chronic urinary incontinence, n% | 4 (0.20) | 1 (0.11) | 0 | 3 (0.37) | <0.01 |
| Chronic, severe knee or hip pain associated with joint stiffness and reduced range of joint motion, n% | 14 (0.70) | 9 (1.03) | 0 | 5 (0.61) | 0.16 |
| Significant, age-adjusted limitations of mobility and/or other basic Activities of Daily Living, n% | 2 (0.10) | 0 | 0 | 2 (0.24) | 0.23 |

**Supplementary table S3.** Baseline characteristics of participants. Note: Data are presented number (percentage). Abbreviations: DVT, deep vein thrombosis; NAFLD, Non-alcoholic fatty liver disease.

| **Table S4 Association of Obesity Status and Arterial Stiffness** | | | | | | | | |
| --- | --- | --- | --- | --- | --- | --- | --- | --- |
|  | **Group** | **No. of cases/Total** | **Model 1** | | **Model 2** | | **Model 3** | |
|  |  |  | **OR (95%CI)** | ***P*** | **OR (95%CI)** | ***P*** | **OR (95%CI)** | ***P*** |
| **Obesity Status** | Non-obesity | 434/856 | 1 |  | 1 |  | 1 |  |
|  | Preclinical obesity | 135/317 | 0.59 (0.45-0.78) | <0.01 | 0.65 (0.48-0.87) | <0.01 | 0.59 (0.44-0.80) | <0.01 |
|  | Clinical obesity | 193/818 | 2.09 (1.70-2.56) | <0.01 | 2.13 (1.71-2.65) | <0.01 | 1.93 (1.54-2.43) | <0.01 |

**Supplementary table S4. Association of Obesity Status and Arterial Stiffness.**

Note: Ordinal Logistic Regression Analysis is used to investigate the association of Obesity Status and Pulse wave.
Model 1: adjusted for age and gender;
Model 2: adjusted for variables in Model 1 plus smoking (yes or no), drinking (yes or no), Physical exercise (yes or no);
Model 3: adjusted for variables in Model 2 plus LDL, (mmol/L), HCY (umol/L), CRP (mg/L), Hypoglycemic treatment (yes or no), Lipid-lowering treatment (yes or no);Abbreviations: OR, Odds Ratio; CI, confidence interval.

| **Table S5 Association of Arterial Stiffness and Cerebral Small Vessel Disease Burden** | | | | | | | | |
| --- | --- | --- | --- | --- | --- | --- | --- | --- |
|  | **Group** | **No. of cases/Total** | **Model 1** | | **Model 2** | | **Model 3** | |
|  |  |  | **OR (95%CI)** | ***P*** | **OR (95%CI)** | ***P*** | **OR (95%CI)** | ***P*** |
| **Obesity Status** | Non-AS | 229/543 | 1 |  | 1 |  | 1 |  |
|  | Moderate-AS | 439/578 | 1.33 (0.96-1.84) | 0.09 | 1.29 (0.91-1.82) | 0.16 | 1.24 (0.87-1.78) | 0.24 |
|  | Severe-AS | 17/262 | 3.38 (1.29-4.41) | <0.01 | 2.18 (1.12-4.24) | 0.02 | 2.03 (1.04-4.17) | 0.04 |

**Supplementary table S4.** Association of Arterial Stiffness and Cerebral Small Vessel Disease Burden.

Note: Binomial Logistic Regression Analysis is used to investigate the association of Pulse wave and Cerebral Small Vessel Disease Burden.
Model 1: adjusted for age and gender;
Model 2: adjusted for variables in Model 1 plus smoking (yes or no), drinking (yes or no), Physical exercise (yes or no);
Model 3: adjusted for variables in Model 2 plus LDL, (mmol/L), HCY (umol/L), CRP (mg/L), Hypoglycemic treatment (yes or no), Lipid-lowering treatment (yes or no).

| **Table S6 Association of Obesity Status combine with clinical symptoms and Cerebral Small Vessel Disease Burden** | | | | | | | | |
| --- | --- | --- | --- | --- | --- | --- | --- | --- |
|  | **Group** | **No. of cases/Total** | **Model 1** | | **Model 2** | | **Model 3** | |
|  |  |  | **OR (95%CI)** | ***P*** | **OR (95%CI)** | ***P*** | **OR (95%CI)** | ***P*** |
| **One** | Non-obesity | 506/873 | 1 |  | 1 |  | 1 |  |
|  | Clinical obesity | 411/259 | 1.51 (1.10-2.08) | 0.01 | 1.52 (1.10-2.09) | 0.01 | 1.44 (1.03-2.01) | 0.03 |
| **Two and more** | Non-obesity | 506/873 | 1 |  | 1 |  | 1 |  |
|  | Clinical obesity | 241/292 | 2.04 (1.33-3.13) | 0.01 | 2.00 (1.30-3.07) | <0.01 | 1.80 (1.13-2.85) | 0.01 |

**Supplementary table S6.** Association of Obesity Status combine with clinical symptoms and Cerebral Small Vessel Disease Burden.

Note: Binomial Logistic Regression Analysis is used to investigate the association of Pulse wave and Cerebral Small Vessel Disease Burden.
Model 1: adjusted for age and gender;
Model 2: adjusted for variables in Model 1 plus smoking (yes or no), drinking (yes or no), Physical exercise (yes or no);
Model 3: adjusted for variables in Model 2 plus LDL, (mmol/L), HCY (umol/L), CRP (mg/L), Hypoglycemic treatment (yes or no), Lipid-lowering treatment (yes or no).

| **Table S7 Association of Obesity Status and Moderate-Severe Cerebral Small Vessel Disease Burden** | | | | | | | | |
| --- | --- | --- | --- | --- | --- | --- | --- | --- |
|  | **Group** | **No. of cases/Total** | **Model 1** | | **Model 2** | | **Model 3** | |
|  |  |  | **OR (95%CI)** | ***P*** | **OR (95%CI)** | ***P*** | **OR (95%CI)** | ***P*** |
| **Obesity Status** | Non-obesity | 211/856 | 1 |  | 1 |  | 1 |  |
|  | Preclinical obesity | 61/317 | 0.67 (0.46-0.98) | 0.04 | 0.67 (0.46-0.98) | 0.04 | 0.72 (0.49-1.06) | 0.10 |
|  | Clinical obesity | 379/818 | 1.76 (1.37-2.25) | <0.01 | 1.76 (1.37-2.26) | <0.01 | 1.64 (1.26-2.13) | <0.01 |

**Supplementary table S7.** Association of Obesity Status and Moderate-Severe Cerebral Small Vessel Disease Burden

Note: Binomial Logistic Regression Analysis is used to investigate the association of Pulse wave and Cerebral Small Vessel Disease Burden.
Model 1: adjusted for age and gender;
Model 2: adjusted for variables in Model 1 plus smoking (yes or no), drinking (yes or no), Physical exercise (yes or no);
Model 3: adjusted for variables in Model 2 plus LDL, (mmol/L), HCY (umol/L), CRP (mg/L), Hypoglycemic treatment (yes or no), Lipid-lowering treatment (yes or no).

| **Table 8. Prevalence of cSVD markers and total cSVD burden by clinical obesity status** | | | | |
| --- | --- | --- | --- | --- |
| **Characteristics** | **Total**  **(n=2011)** | **Non-obesity**  **(n=873)** | **Preclinical obesity**  **(n=317)** | **Clinical obesity**  **(n=821)** |
| Total cSVD burden, n (%) |  |  |  |  |
| 0 | 689 (34.26) | 367 (18.25) | 153 (7.61) | 169 (8.40) |
| 1 | 652 (32.42) | 289 (14.37) | 101 (5.02) | 262 (13.03) |
| 2 | 378 (18.80) | 128 (6.36) | 42 (2.09) | 208 (10.34) |
| 3 | 168 (8.35) | 58 (2.88) | 14 (0.70) | 96 (4.77) |
| 4 | 124 (6.17) | 31 (1.54) | 6 (0.30) | 87 (4.33) |
| cSVD (≥1 point) , n (%) | 1322 (65.74) | 506 (25.16) | 163 (8.11) | 653 (32.47) |
| The imaging markers of cSVD, n (%) |  |  |  |  |
| WMH | 439 (21.83) | 143 (7.11) | 44 (2.19) | 252 (12.53) |
| LA | 277 (13.77) | 82 (9.39) | 17 (5.36) | 178 (21.68) |
| CMB | 506 (25.12) | 193 (9.60) | 45 (2.24) | 268 (13.33) |
| PVS | 1183 (58.83) | 423 (21.03) | 144 (7.16) | 616 (30.63) |
| **Supplementary table S8.** Prevalence of cSVD markers and total cSVD burden by clinical obesity status  Note: cSVD, cerebral small vessel disease; WMH, white matter hyperintensities; LA,lacunar infarcts; CMB, cerebral microbleeds ;PVS, enlarged perivascular spaces. | | | | |

| **Supplementary Table 9 Comparison of baseline characteristics between original complete cases and imputed dataset** | | |
| --- | --- | --- |
| Variable | Original complete cases | Imputed dataset |
| Smoking history, n% | 235 (29.06) | 587 (29.19) |
| Drinking history, n% | 669 (36.40) | 728 (36.20) |
| Physical exercise, n% | 1036 (57.65) | 1154 (57.38) |
| LDL-C, mmol/L | 3.12 ± 0.78 | 3.13 ± 0.79 |
| HCY, μmol/L | 12.49 (10.20-15.70) | 12.49 (10.22-15.86) |
| CRP, mg/L | 1.20 (0.60-2.20) | 1.20 (0.60-2.30) |
| **Supplementary Table 9.** Comparison of baseline characteristics between original complete cases and imputed dataset | | |
